# The anti-cancer drug dabrafenib is not cardiotoxic and inhibits cardiac remodelling and fibrosis in a murine model of hypertension

**DOI:** 10.1042/CS20210192

**Published:** 2021-07-23

**Authors:** Daniel N. Meijles, Joshua J. Cull, Susanna T.E. Cooper, Thomais Markou, Michelle A. Hardyman, Stephen J. Fuller, Hajed O. Alharbi, Zoe H.R. Haines, Viridiana Alcantara-Alonso, Peter E. Glennon, Mary N. Sheppard, Peter H. Sugden, Angela Clerk

**Affiliations:** 1 School of Biological Sciences, University of Reading, Reading RG6 2AS, U.K.; 2 Molecular and Clinical Sciences Institute, St George’s University of London, London SW17 0RE, U.K.; 3 University Hospitals Coventry and Warwickshire, University Hospital Cardiology Department, Clifford Bridge Road, Coventry CV2 2DX, U.K.; 4 CRY Cardiovascular Pathology Department, St. George's University of London, London, U.K.; 5 St. George’s Healthcare NHS Trust, London, U.K.

**Keywords:** cardio oncology, fibrosis, hypertension, myocardial remodeling, Raf kinase

## Abstract

Raf kinases signal via extracellular signal-regulated kinases 1/2 (ERK1/2) to drive cell division. Since activating mutations in BRAF (B-Raf proto-oncogene, serine/threonine kinase) are highly oncogenic, BRAF inhibitors including dabrafenib have been developed for cancer. Inhibitors of ERK1/2 signalling used for cancer are cardiotoxic in some patients, raising the question of whether dabrafenib is cardiotoxic. In the heart, ERK1/2 signalling promotes not only cardiomyocyte hypertrophy and is cardioprotective but also promotes fibrosis. Our hypothesis is that ERK1/2 signalling is not required in a non-stressed heart but is required for cardiac remodelling. Thus, dabrafenib may affect the heart in the context of, for example, hypertension. In experiments with cardiomyocytes, cardiac fibroblasts and perfused rat hearts, dabrafenib inhibited ERK1/2 signalling. We assessed the effects of dabrafenib (3 mg/kg/d) on male C57BL/6J mouse hearts *in vivo*. Dabrafenib alone had no overt effects on cardiac function/dimensions (assessed by echocardiography) or cardiac architecture. In mice treated with 0.8 mg/kg/d angiotensin II (AngII) to induce hypertension, dabrafenib inhibited ERK1/2 signalling and suppressed cardiac hypertrophy in both acute (up to 7 d) and chronic (28 d) settings, preserving ejection fraction. At the cellular level, dabrafenib inhibited AngII-induced cardiomyocyte hypertrophy, reduced expression of hypertrophic gene markers and almost completely eliminated the increase in cardiac fibrosis both in interstitial and perivascular regions. Dabrafenib is not overtly cardiotoxic. Moreover, it inhibits maladaptive hypertrophy resulting from AngII-induced hypertension. Thus, Raf is a potential therapeutic target for hypertensive heart disease and drugs such as dabrafenib, developed for cancer, may be used for this purpose.

## Introduction

The extracellular signal-regulated kinase 1/2 (ERK1/2) cascade promotes cell cycle entry and cell division in proliferating cells [1]. In the heart, ERK1/2 signalling promotes cardiomyocyte hypertrophy and, independently of this, is generally cardioprotective [2,3]. Activation of ERK1/2 requires phosphorylation by mitogen-activated protein kinase kinases 1/2 (MKK1/2), which are phosphorylated/activated by the upstream Raf kinases (ARAF, BRAF, RAF1) or (in inflammation) Cot/Tpl2 [4,5]. Activation of Raf kinases requires interaction with activated, GTP-bound Ras, which brings the enzyme to the membrane for activation by phosphorylation [4]. Raf kinases operate as homo- or heterodimers. They also require phosphorylation of specific residues to increase activity (e.g. Ser338/Ser341 in human RAF1) plus dephosphorylation of other residues to permit activation (e.g. Ser43 in human RAF1).

Mutations that activate ERK1/2 signalling cause cancer. Activating mutations in BRAF are particularly prevalent, being associated with ∼30% of all cancers and ∼60% of melanomas [6,7]. As such, efficacious BRAF inhibitors (e.g. dabrafenib [8]) have been developed for clinical use. Whilst these inhibitors were designed to target oncogenic BRAF, they also inhibit wild-type Raf kinases, and dabrafenib has IC_50_ values of 5.2 and 6.3 nM for BRAF and RAF1, respectively [8]. The first generation of drugs to be developed are competitive inhibitors acting at the ATP-binding site (i.e. Type 1 or Type 1.5 inhibitors), and these can lock the enzyme in an active conformation. This led to the discovery of the ‘Raf paradox’ [9], where non-saturating concentrations of drugs can activate ERK1/2 signalling rather than inhibit the pathway. However, not all Raf inhibitors have paradox-inducing abilities, and little is known about their effects in ‘normal’ cardiac cells, so it is difficult to predict how Raf inhibitors such as dabrafenib affect the heart.

Hypertensive heart disease is a major cause of morbidity and mortality worldwide [10]. Elevated blood pressure induces an initial adaptive response allowing the heart to maintain cardiac output resulting from an increased workload [11]. Terminally differentiated contractile cardiomyocytes become hypertrophied (increase in size), with increases in and adaptation of the myofibrillar apparatus. Over time, however, this adaptative response is unsustainable and contractile function becomes compromised, leading to heart failure. At a cellular level, the switch to failure includes increased cardiomyocyte cell death [12], myocardial inflammation and increased deposition of fibrotic material [13]. While strategies exist to combat elevated blood pressure (e.g. angiotensin-converting enzyme inhibitors [14]), strategies to reduce cardiomyocyte cell death, improve cardiac contractility and reduce fibrosis are urgently needed to treat hypertensive heart failure [15].

ARAF, BRAF and RAF1 are all expressed in human and murine hearts [16]. In cultured rodent cardiomyocytes, RAF1 and ARAF are activated by hypertrophic stimuli such as endothelin-1 [17,18]. Mice with cardiomyocyte-specific expression of a dominant-negative form of RAF1 and subjected to cardiac pressure-overload exhibit increased cardiomyocyte apoptosis resulting in enhanced cardiomyopathy [19,20], consistent with reduced cardioprotection. The role of BRAF in these processes is even less well understood. Here, we investigated the effects of dabrafenib (a Type 1.5 Raf inhibitor) on the heart to determine whether it is likely to be cardiotoxic in hypertension, or if it may protect against hypertensive heart disease. We established that: (1) Raf paradox signalling induced by dabrafenib in cardiac cells is limited; (2) dabrafenib inhibits ERK1/2 activity in isolated cardiac cells, in *ex vivo* perfused adult rat hearts and in a murine model of hypertension induced by angiotensin II (AngII); and (3), inhibition of Raf kinases with dabrafenib reduced cardiomyocyte hypertrophy, cardiac inflammation and cardiac fibrosis induced by AngII in both acute (7 d) and chronic (28 d) treatment conditions. Thus, dabrafenib and, possibly, other Raf kinase inhibitors may be therapeutically useful for treating hypertensive heart disease.

## Materials and methods

### Ethical statement for animal experiments

Procedures were performed in accordance with the European Parliament Directive 2010/63/EU on the protection of animals used for scientific purposes, with local institutional animal care committee procedures (University of Reading) and the U.K. Animals (Scientific Procedures) Act 1986. C57Bl/6J mice, Sprague-Dawley rats and Alzet osmotic minipumps were from Charles River U.K. Details of animal housing, husbandry and welfare are provided in [21].

### *In vivo* mouse studies

Wild-type male (7 wk) C57Bl/6J mice were imported into the BioResource Unit at University of Reading and allowed to acclimatize for 2 weeks before experimentation. Mice were randomly allocated to each treatment group; body weights are provided in Supplementary Table S1. Drug delivery used Alzet osmotic pumps (models 1007D or 1004), filled according to the manufacturer’s instructions. Mice received minipumps for delivery of 0.8 mg/kg/d angiotensin II (AngII; Merck) or vehicle (acidified PBS) without/with DMSO/PEG mix [50% (v/v) dimethyl sulphoxide (DMSO), 20% (v/v) polyethylene glycol 400, 5% (v/v) propylene glycol, 0.5% (v/v) Tween 80] or 3 mg/kg/d dabrafenib (Selleck Chemicals) dissolved in DMSO/PEG mix. Separate minipumps were used for AngII and dabrafenib delivery. Minipumps were incubated overnight in sterile PBS (37°C), then implanted subcutaneously under continuous inhalation anaesthesia using isoflurane (induction at 5%, maintenance at 2–2.5%) mixed with 2 l/min O_2_, as previously described [22].

Echocardiography was performed with a Vevo 2100 imaging system using a MS400 18-38 MHz transducer (Visualsonics) as previously described [22]. Left ventricular cardiac dimensions were assessed from short axis M-mode images with the axis placed at the mid-level of the left ventricle at the level of the papillary muscles. Cardiac function for acute treatments (up to 7 d) was assessed from these images on the basis that the stresses on the hearts are likely to be relatively uniform resulting in minimal deformation of the heart. Thus, the algorithms used are appropriate for comparative data. Data analysis was performed using VevoLAB software (Visualsonics) by independent assessors blinded to intervention. Data were gathered from two M-mode scans at each time point, taking mean values across at least three cardiac cycles for each echocardiogram. The diameter of the aorta was measured with the calliper function from B-mode images at the end of cardiac systole (with the aorta at its widest) and following aortic contraction, taking an average of measurements across three cardiac cycles. Cardiac function and global longitudinal strain were measured in studies with 28 d treatments from B-mode long axis images using Vevo Strain software for speckle tracking. Global circumferential strain was measured using B-mode short axis images.

Mice were killed by CO_2_ inhalation followed by cervical dislocation. Hearts were excised quickly, washed in PBS, blotted to remove excess PBS and snap-frozen in liquid N_2_ or fixed in 10% buffered formalin for histology.

### Histology and assessment of myocyte size and fibrosis

Histological staining and analysis were performed as previously described [22], assessing general morphology by haematoxylin and eosin (H&E) and fibrosis by Masson’s trichrome and picrosirius red (PSR). Sections for the study of the effects of dabrafenib on AngII-induced cardiac pathology over 28 d were prepared and stained by HistologiX Limited. Analysis was performed by independent assessors blinded to treatment groups.

### Adult rat heart perfusions

Adult male (300–350 g) Sprague-Dawley rats were used for heart perfusions. Hearts were prepared and perfused in the Langendorff mode as described in [21]. Hearts were perfused for 15 min with Krebs-Henseleit bicarbonate-buffered saline (25 mM NaHCO_3_, 119 mM NaCl, 4.7 mM KCl, 2.5 mM CaCl_2_, 1.2 mM MgSO_4_, 1.2 mM KH_2_PO_4_, pH 7.4, containing 10 mM glucose and equilibrated with 95% O_2_/5% CO_2_) without or with dabrafenib (5 µM) or trametinib (1 µM). Dabrafenib and trametinib were from Selleck Chemicals. Perfusions were continued for 10 min without/with addition of human FGF2 (0.5 µg/ml; Cell Guidance Systems Ltd., U.K.). Hearts were ‘freeze-clamped’ between aluminium tongs cooled in liquid nitrogen and pulverized under liquid N_2_. Heart powders were stored at −80°C.

### Cell cultures

Neonatal rat cardiomyocytes were prepared and cultured from 2 to 4 d Sprague-Dawley rats as described previously [23]. Human cardiac fibroblasts (PromoCell) were grown in Fibroblast growth medium-3 (FGM3, PromoCell). Fibroblasts were seeded the day before experimentation (at a density to achieve 90% confluence after 24 h) and synchronized overnight in M199 medium containing 0.1% (v/v) foetal calf serum and 100 U/ml penicillin and streptomycin. Cells were exposed to the concentrations of dabrafenib and for the times indicated prior to harvesting for immunoblotting.

### RNA preparation and qPCR

Total RNA was prepared using RNA Bee (AMS Biotechnology Ltd) with 1 ml per 4 × 10^6^ cardiomyocytes or 10–15 mg mouse heart powder as previously published [23]. Quantitative PCR (qPCR) was performed as previously described [23], using primers from Invitrogen by Thermo Fisher Scientific. Details of primer sequences are provided in Supplementary Table S2. *Gapdh* was the reference gene for the study, with relative quantification obtained using the Δ*C*t (threshold cycle) method; relative expression was calculated as 2^−ΔΔCt^ and normalized to vehicle.

### Sample preparation and immunoblotting

Cells and heart powders were prepared for immunoblotting as published [22], with protein concentrations for equal loading determined by BioRad Bradford assay using bovine serum albumin standards. Proteins were separated by SDS-PAGE on 10% (w/v) polyacrylamide resolving gels with 6% stacking gels and transferred electrophoretically to nitrocellulose using a BioRad semi-dry transfer cell (10 V, 60 min) as described [23]. Proteins were detected using antibodies (1/1000 dilution) to phosphorylated or total ERK1/2 (Cell Signaling Technologies; Cat. Nos. 4377 and 4695, respectively) or Gapdh (Cell Signaling Technologies: Cat. No. 2118). Bands were detected by enhanced chemiluminescence using ECL Prime Western Blotting detection reagents with visualization using an ImageQuant LAS4000 system (GE Healthcare). ImageQuant TL 8.1 software (GE Healthcare) was used for densitometric analysis of the bands.

### Image processing

Images were cropped and reorientated for presentation using Photoshop CC then resized in Adobe Illustrator, maintaining the original proportions and using the same resizing factor for all images within a Figure.

### Statistical analysis

Data analysis was performed using Microsoft Excel and GraphPad Prism. Statistical analysis was performed using GraphPad Prism with two-tailed unpaired *t* tests, two-tailed one-way ANOVA or two-tailed two-way ANOVA as indicated. A multiple comparison test was used in combination with ANOVA as indicated in the figure legends. Graphs were plotted with GraphPad Prism 9.0.

## Results

### Dabrafenib inhibits ERK1/2 signalling in cardiac cells and perfused adult rat hearts

**Figure 1:**
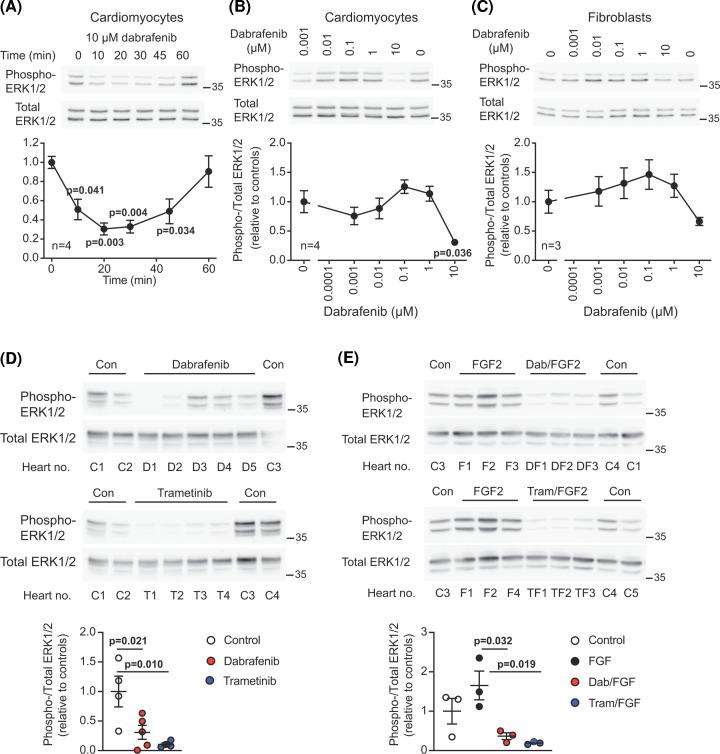
Dabrafenib inhibits ERK1/2 signalling in the heart Neonatal rat cardiomyocytes or human cardiac fibroblasts (as indicated) were exposed to 10 µM dabrafenib for the times indicated (**A**), or to the indicated concentrations of dabrafenib for 20 min (**B** and **C**). Adult male rat hearts were perfused in Langendorff-mode under basal conditions (**D**) or with 0.5 µg/ml FGF2 (**E**) without/with 5 µM dabrafenib (Dab) or 1 µM trametinib (Tram) as indicated. Proteins (extracts from 0.2 × 10^6^ cells for cardiomyocytes; 20 µg for fibroblasts; 40 µg for hearts) were immunoblotted for phosphorylated (phospho-) or total ERK1/2. Representative blots are shown in the upper panels with positions of relative molecular mass markers on the right of each image. Densitometric analysis is provided in the lower panels. Results are provided as means ± SEM for *n* = 3 or 4 independent cell preparations of hearts as indicated. For (**D** and **E**), individual data points are also shown and individual hearts are labelled for direct comparison (C, control; D, dabrafenib; DF, Dab+FGF2; F, FGF2; T, trametinib; TF, Tram+FGF2). Statistical analysis used one-way ANOVA with Holm-Sidak's post-test. In (**A** and** B**), significance is given relative to samples with no dabrafenib.

Dabrafenib is a Type 1.5 Raf inhibitor that can activate (rather than inhibit) ERK1/2 signalling via the ‘Raf paradox’ in some cancer cells [9]. We first assessed if dabrafenib activates Raf signalling in primary cardiac cells (neonatal rat cardiomyocytes; human cardiac fibroblasts) *in vitro*. In cardiomyocytes, 10 µM dabrafenib inhibited basal ERK1/2 phosphorylation (i.e. activation), with maximal inhibition at ∼20 min (Figure 1A). Inhibition was sustained to ∼40 min, but by 60 min the inhibitory effect was lost. This may be due to compensatory mechanisms or drug instability in the conditions used. The concentration-dependency of the response was assessed at 20 min. ERK1/2 phosphorylation was inhibited by 10 µM dabrafenib in both cell types (Figure 1B,C), with limited effects (activating or inhibitory) at lower doses. We next determined if dabrafenib affects ERK1/2 signalling in intact Langendorff perfused adult male rat hearts, comparing dabrafenib with trametinib, a MKK1/2 inhibitor [24]. Dabrafenib (5 µM) and trametinib (1 µM) each inhibited basal ERK1/2 phosphorylation (Figure 1D) and agonist-induced activation of ERK1/2 in hearts perfused with FGF2 (Figure 1E). In conclusion, dabrafenib has limited ability to induce Raf paradox signalling in primary cardiac cells, but inhibits at higher concentrations.

### Dabrafenib alone does not significantly affect cardiac function, dimensions or architecture *in vivo*

Since ERK1/2 pathway modulators can cause adverse cardiac events in cancer patients [25], and our data suggest that it inhibits basal ERK1/2 signalling in the heart (Figure 1), we next assessed whether dabrafenib alone has any cardiac effects at baseline in animals *in vivo*. In cancer patients, the recommended dose of dabrafenib is 150 mg, twice daily [26]. With a body weight of 60–100 kg, the dosage would be 3–5 mg/kg/d. We selected a dose at the lower end of this spectrum and male C57Bl/6J mice were implanted with osmotic minipumps for delivery of vehicle or 3 mg/kg/d dabrafenib. The effects on cardiac function/dimensions were assessed by echocardiography. This concentration of dabrafenib had no significant effect on any cardiac parameter studied, whether associated with function (heart rate, cardiac output, ejection fraction, fractional shortening) or dimensions [e.g. left ventricular (LV) wall thickness or internal diameter] (Supplementary Figure S1A and Supplementary Table S3). Dabrafenib also had no effect on cardiac architecture assessed using standard histological staining methods, or any significant effects on mRNA expression of hypertrophic marker genes (*Myh7, Nppa* and *Nppb*) assessed by qPCR (Supplementary Figure S1B,C). These findings provide further evidence that dabrafenib is not overtly cardiotoxic and has no immediate adverse cardiac effects in unstressed hearts.

### Dabrafenib inhibits acute hypertensive cardiac remodelling induced by AngII in mice *in vivo*

**Figure 2:**
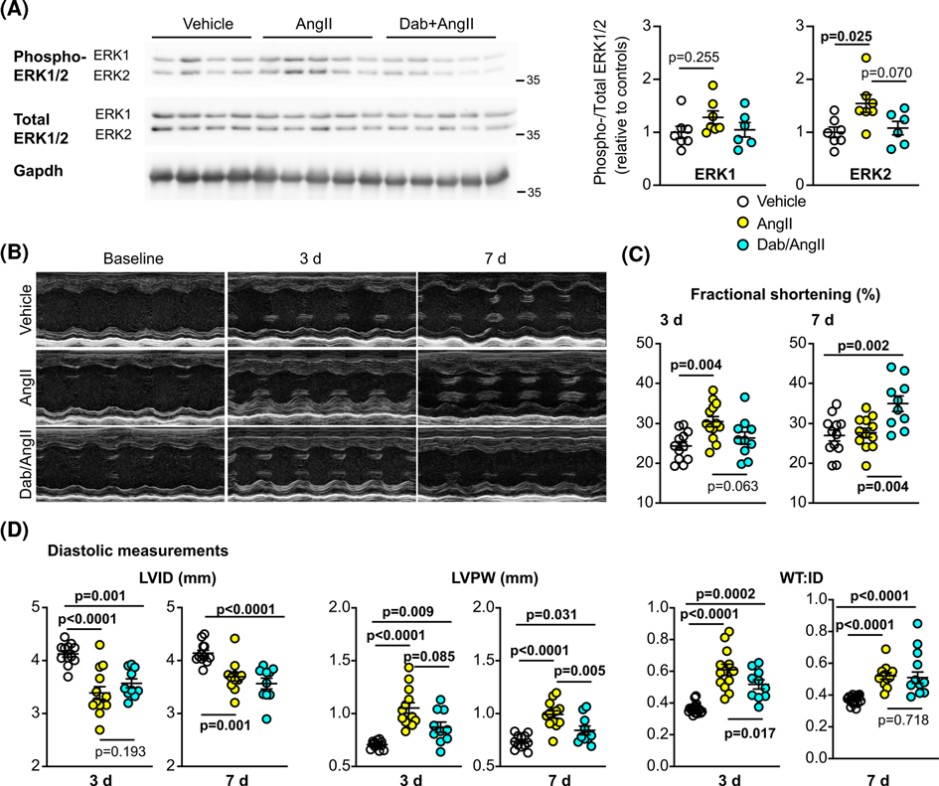
Dabrafenib inhibits cardiac hypertrophy induced in mice *in vivo* by acute treatment with AngII C57BL/6J male mice were treated with vehicle, 0.8 mg/kg/d AngII or 3 mg/kg/d dabrafenib (Dab) with AngII for up to 7 d. (**A**) Mice were treated for 24 h. Total proteins were extracted from the hearts and 25 µg immunoblotted for phosphorylated (phospho-) ERK1/2, total ERK1/2 or Gapdh. Representative immunoblots are on the left with densitometric analysis on the right. (**B**) Cardiac function and dimensions were assessed using echocardiography. Representative short axis M-mode echocardiograms are shown at baseline and at 3 and 7 d post-treatment. Quantitative assessment of echocardiograms are provided for fractional shortening (**C**) and diastolic cardiac dimensions (**D**). All echocardiography data are provided in Supplementary Table S3. LV, left ventricle; ID, internal diameter; PW, posterior wall; WT, wall thickness (anterior wall + posterior wall). Quantification data are individual points with means ± SEM. Individual *P* values are shown (one-way ANOVA with Holm-Sidak’s post-test).

Although our data with dabrafenib at baseline suggest Raf signalling is not required for maintenance of normal cardiac function, this may be different in the hypertensive heart that undergoes remodelling to maintain function. We therefore treated male C57Bl/6J mice with AngII (0.8 mg/kg/d) to induce hypertension and remodelling as in [22], in the absence or presence of 3 mg/kg/d dabrafenib, initially assessing the effects over 7 d. AngII (24 h) increased cardiac ERK1/2 phosphorylation (i.e. activity) with a significant increase in ERK2 phosphorylation, an effect that was absent with dabrafenib (Figure 2A). We assessed the effects on cardiac dimensions and function at 3 and 7 d by echocardiography (Figure 2B–D; Supplementary Table S3). AngII increased fractional shortening at 3 d, but this was normalized by 7 d (Figure 2C). Dabrafenib inhibited these increases at 3 d, but enhanced the response at 7 d. AngII also significantly increased diastolic and systolic LV wall thickness (WT) as early as 3 d, with decreased LV internal diameter (ID), resulting in significant increase in the WT:ID ratio (Figure 2D). This is consistent with an early compensatory hypertrophy associated with pressure-overload [27]. Dabrafenib significantly inhibited this response. The increase in wall thickness induced by AngII was sustained through to 7 d, but the change in LVID started to normalize so the WT:ID ratio was reduced relative to 3 d, suggesting that the heart was starting to adapt. Dabrafenib still inhibited the increase in wall thickness induced by AngII at 7 d and the WT:ID ratio remained stable.

**Figure 3:**
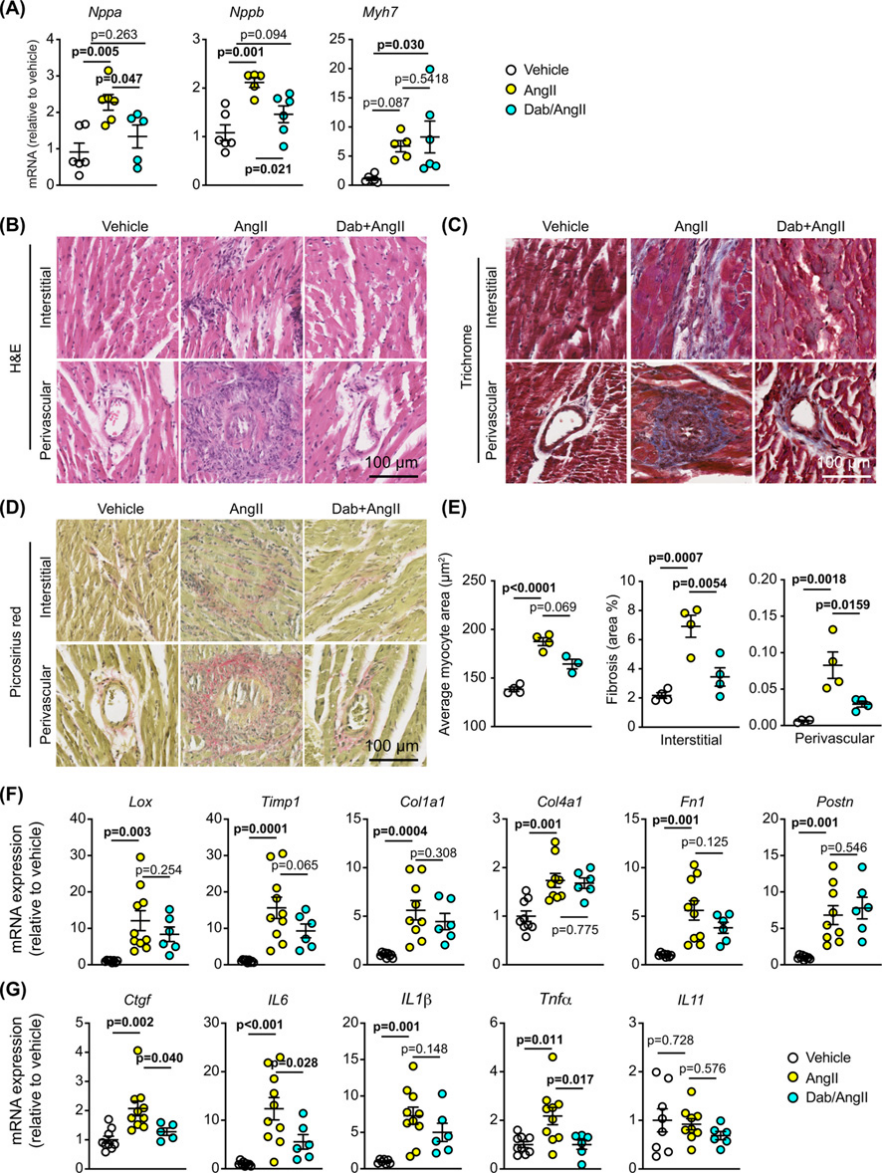
Dabrafenib reduces cardiac fibrosis induced in mice *in vivo* by acute treatment with AngII C57BL/6J male mice were treated with vehicle, 0.8 mg/kg/d AngII or 3 mg/kg/d dabrafenib (Dab) with AngII for 7 d. (**A**) mRNA expression of *Nppa, Nppb* and *Myh7* in mouse hearts was assessed by qPCR. (**B**) Cardiomyocyte size was assessed using H&E staining (B) with quantification provided in (E) (left panel). Cardiac fibrosis was assessed using trichrome (**C**) and picrosirius red (**D**), with quantification provided in (**E**) (right panels). mRNA expression in mouse hearts of markers of fibrosis (**F**) or pro-inflammatory cytokines or pro-fibrotic factors (**G**) was assessed by qPCR. Quantification data are individual points with means ± SEM. Individual *P* values are shown (one-way ANOVA with Holm-Sidak’s post-test).

To understand the effects of dabrafenib on AngII-induced changes in the heart, we undertook detailed assessment of the changes in gene expression and architecture of the heart after 7 d treatment with AngII. Dabrafenib significantly inhibited the increased cardiac mRNA expression of *Nppa* and *Nppb* induced by AngII at 7 d (Figure 3A), although it did not affect the increase in expression of *Myh7*. At a cellular level, AngII increased cardiomyocyte hypertrophy (with increased cross-sectional area) and cardiac fibrosis (Figure 3B–E). Dabrafenib significantly inhibited AngII-induced cardiomyocyte hypertrophy (Figure 3B,E), and almost eliminated both perivascular and interstitial fibrosis (Figure 3C–E). These effects most likely account for the reduction in cardiac hypertrophy and enhanced function detected by echocardiography (Figure 2C,D; Supplementary Table S3). The increase in expression of mRNAs for fibrosis-associated enzymes (*Lox, Timp1*) and extracellular matrix genes (*Col1a1, Col4a1, Fn1, Postn*) induced by AngII were not significantly inhibited by dabrafenib, although there was a trend to a reduced expression (Figure 3F). In contrast, dabrafenib significantly inhibited the increase in mRNAs encoding proinflammatory cytokines and profibrotic factors (*Ctgf, IL6, IL1β, Tnfα, IL11*) induced by AngII at 7 d (Figure 3G). This suggests that one mechanism for the maintenance of cardiac function in dabrafenib-treated hypertensive mice is via reduced myocardial inflammation that potentially impacts on fibrosis and, subsequently, cardiomyocyte hypertrophy. Overall, these studies indicate that dabrafenib moderates cardiac hypertrophy induced acutely by hypertension, with a predominant overall effect on cardiac fibrosis.

### Dabrafenib inhibits hypertensive cardiac remodelling in chronic AngII-infused mice *in vivo*

**Figure 4:**
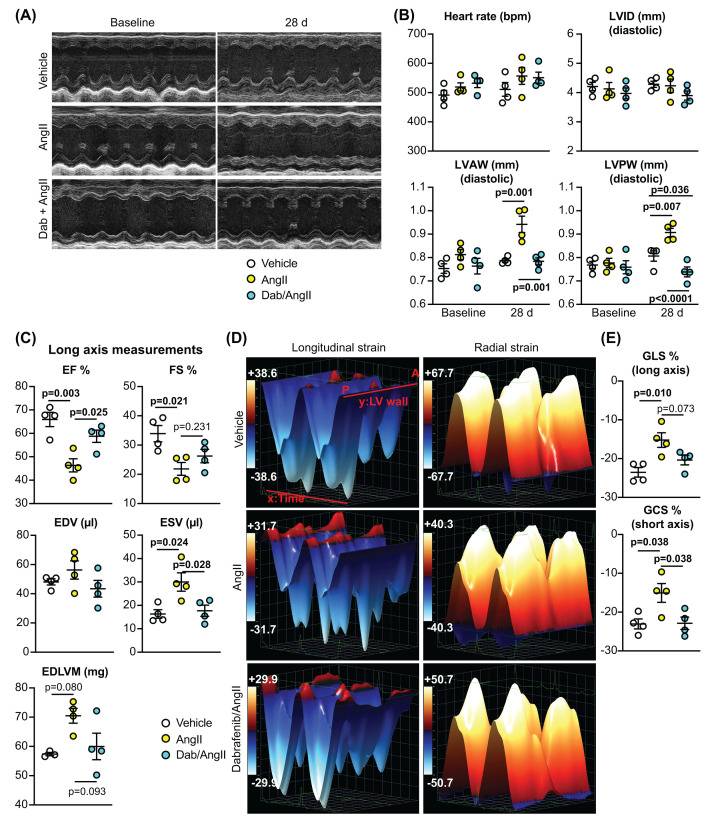
Dabrafenib inhibits changes in cardiac dimensions and function induced in mouse hearts by AngII over 28 dP C57BL/6J male mice were treated with vehicle, 0.8 mg/kg/d AngII or 3 mg/kg/d dabrafenib with AngII (28 d). Cardiac function and dimensions were assessed using echocardiography. (**A**) Representative short axis M-mode echocardiograms from animals at baseline and with 28 d treatment. (**B**) Quantitative assessment of M-mode echocardiograms showing heart rate and dimensions; AW, anterior wall; LV, left ventricle; ID, internal diameter. All echocardiography data are provided in Supplementary Table S5. (**C**) Quantification of strain data for B-mode long axis views; EDV, end diastolic volume; EDLVM, end diastolic left ventricular mass; EF, ejection fraction; ESV, end systolic volume; FS, fractional shortening. (**D**) Representative 3D images showing longitudinal and radial strain assessed from long axis B-mode images. (**E**) Global longitudinal strain (GLS) and global circumferential strain (GCS) were measured from long and short axis B-mode images, respectively. Quantification data show individual points with means ± SEM. Individual *P* values are shown using one-way ANOVA with Holm-Sidak’s post-test in (C and E), and two-way ANOVA with Holm-Sidak’s post-test in (B).

**Figure 5:**
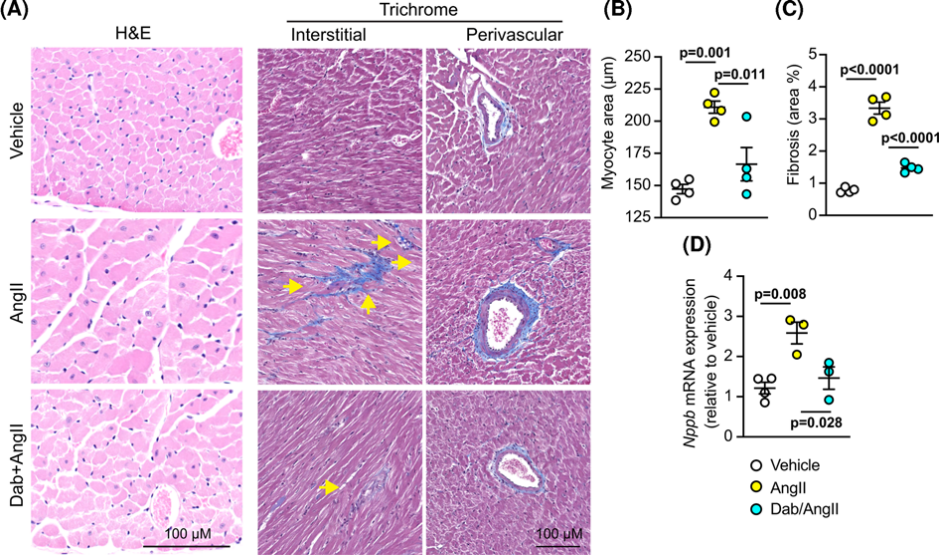
Dabrafenib inhibits cardiomyocyte hypertrophy and cardiac fibrosis induced in mouse hearts by AngII over 28 d C57BL/6J male mice were treated with vehicle, 0.8 mg/kg/d AngII or 3 mg/kg/d dabrafenib with AngII (28 d). (**A**) Representative images from mouse heart sections stained with H&E (left panels) or Masson’s Trichrome (centre and right panels, showing interstitial and perivascular areas, respectively). (**B**) Quantification of cardiomyocyte area from sections stained with H&E. (**C**) Quantification of cardiac fibrosis from sections stained with Trichrome. (**D**) mRNA expression of *Nppb* was measured by qPCR. Quantification data are provided as individual points with means ± SEM. Individual *P* values are shown (one-way ANOVA with Holm-Sidak’s post-test).

Given that dabrafenib inhibited cardiac remodelling and fibrosis in hypertensive mice over 7 d, we next assessed if the effects could be sustained over longer periods. Experiments were conducted in male C57Bl/6J mice treated with 3 mg/kg/d dabrafenib or vehicle in the presence/absence of AngII (0.8 mg/kg/d) for 28 d. Dabrafenib alone had no effect on cardiac dimensions/function over this time (Supplementary Table S4). Treatment with AngII for 28 d resulted in increased LV wall thickness whilst maintaining a similar internal diameter (Figure 4A,B; Supplementary Table S5). The increase in wall thickness was inhibited by dabrafenib. To gain insight into the effects on cardiac function, we used echocardiography with 2D speckle-tracking strain analysis of long axis views of the heart. AngII induced clear systolic dysfunction with reduced ejection fraction and fractional shortening, and increased end systolic (not diastolic) volume (Figure 4C). Though not statistically significant, this was associated with an increase in predicted end diastolic left ventricular mass. Functional measurements were supported by strain data (Figure 4D,E; N.B. global longitudinal and circumferential strain measurements are negative values). AngII caused a reduction in global longitudinal and radial strain (measured from the long axis), a result of reduced cardiac contractility. This was associated with reduced global circumferential strain (measured from the short axis view). Dabrafenib had a normalizing effect on all of these parameters. At the cellular level (Figure 5), as with acute hypertension studies (Figure 3), dabrafenib inhibited the increase in cardiomyocyte cross-sectional area induced by AngII, with a significant reduction in *Nppb* mRNA expression, and in perivascular and interstitial fibrosis.

**Figure 6:**
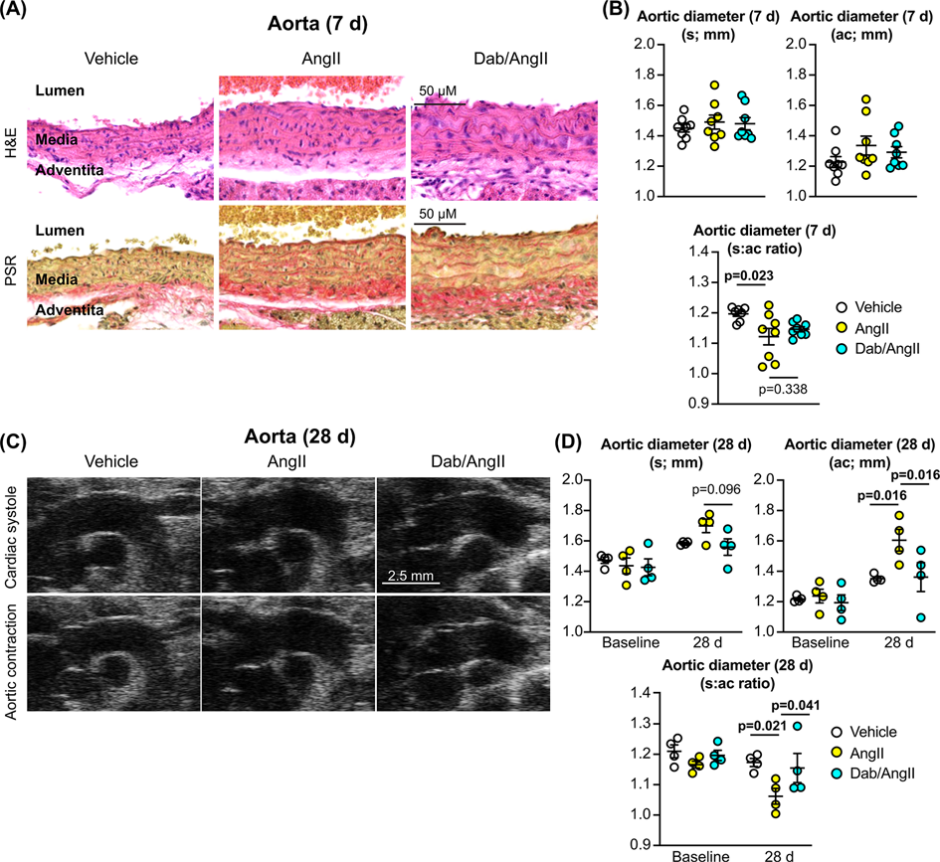
Effects of dabrafenib on the aorta in mice treated with AngII C57BL/6J male mice were treated with vehicle, 0.8 mg/kg/d AngII or 3 mg/kg/d dabrafenib with AngII for 7 or 28 d as indicated. (**A**) The aorta was fixed and stained with H&E or picrosirius red (PSR). Representative images are shown. (**B**) Ultrasound B-mode images of the aorta following 7 d treatment were used to measure the internal diameter at the end of cardiac systole (s) with the aorta at its widest diameter, and following aortic contraction (ac). (**C**) Representative images of the aorta at the end of cardiac systole and following aortic contraction. (**D**) Ultrasound B-mode images of the aorta following 28 d treatment were used to measure the internal diameter. Quantification data are provided as individual points with means ± SEM. Individual *P* values are shown using one-way (B) or two-way (D) ANOVA with Holm-Sidak’s post-test.

In addition to assessing the cardiac effects of dabrafenib on the response to AngII, we examined the effects on the aorta. AngII (7 d) induced an increase in the medial wall thickness, associated with increased fibrosis (Figure 6A). Dabrafenib reduced the degree of fibrosis, but did not suppress the increase in thickness of medial layer suggesting that the overall response of the aorta to hypertensive pressures was retained. The internal diameter of the ascending aorta was measured in ultrasound images, comparing the difference at the end of cardiac systole (when the aorta is most distended) and following aortic contraction. There was no difference in the diameter of the aorta at the end of cardiac systole between vehicle-treated mice and mice treated with AngII with or without dabrafenib over 7 d (Figure 6B). However, the diameter following aortic contraction was larger in mice treated with AngII, resulting in a smaller ratio between the widest and narrowest measurements. This is consistent with reduced flexibility of the aortic wall. By 28 d, the aortae in mice treated with AngII appeared substantially more rigid than those of either the vehicle-treated mice or mice treated with dabrafenib and AngII, with little change in aortic diameter through the cardiac cycle (Figure 6C,D). Thus, dabrafenib may have additional benefits on the heart by preventing hypertension-induced deterioration of the elasticity of the aorta.

## Discussion

**Figure 7:**
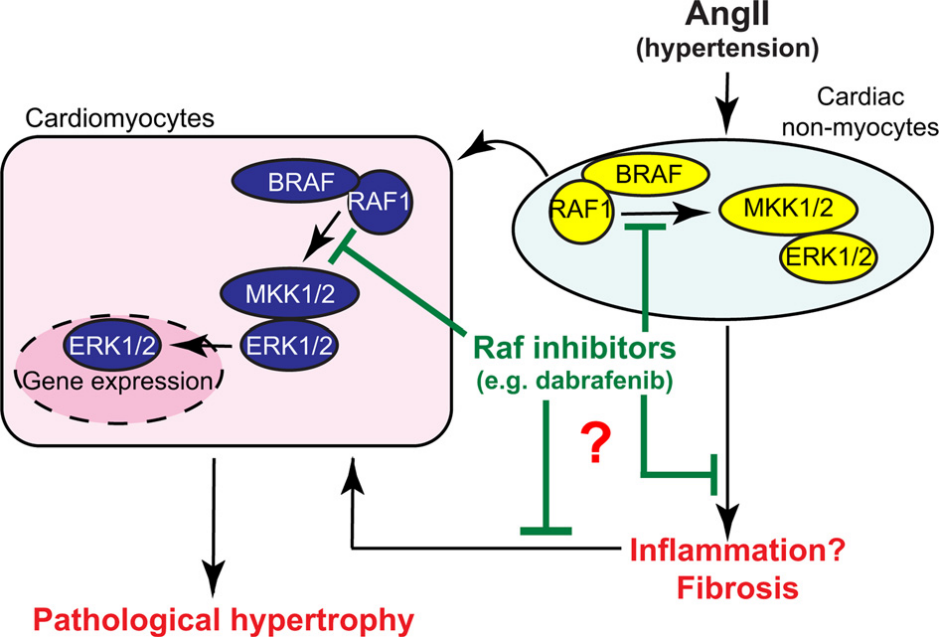
Schematic representation of the conclusions from this study AngII-induced hypertension activates Raf kinases (BRAF and RAF1) in the heart whether directly (particularly in cardiac non-myocytes such as fibroblasts) or indirectly (cardiomyocytes potentially respond to factors released by non-myocytes). Raf kinases signal through the ERK1/2 cascade to promote changes in gene expression and induce phenotypic responses. In cardiomyocytes, this promotes cardiomyocyte hypertrophy to combat increased workload. AngII also increases cardiac inflammation and activates Raf signalling in cardiac non-myocytes promoting cell proliferation and fibrosis. Increased cardiac fibrosis increases the workload on the heart causing further cardiomyocyte hypertrophy. Over time, this leads to pathological remodelling of the hearts that can lead to hypertensive heart disease and failure. Raf inhibitors such as dabrafenib inhibit Raf signalling in both cardiomyocytes and cardiac non-myocytes, reducing cardiac inflammation and fibrosis, reducing cardiomyocyte hypertrophy, and protecting the heart from hypertensive heart disease; ERK, extracellular signal-regulated kinase; MKK, mitogen-activated protein kinase kinase.

The importance of ERK1/2 signalling in promoting cardiomyocyte hypertrophy and cardioprotection has been known for many years [2,3], but the role of ERK1/2 signalling in promoting cardiac fibrosis has emerged more recently [28–30]. This raises the question of whether it is better to inhibit ERK1/2 and prevent fibrosis or if we should activate ERK1/2 to increase cardioprotection and cardiomyocyte hypertrophy. Furthermore, would inhibiting ERK1/2 to prevent fibrosis be detrimental to cardioprotection and hypertrophy, and vice versa? This dilemma is difficult to resolve at the theoretical level and can only be addressed experimentally. Here, as outlined in the schematic in Figure 7, we show that dabrafenib, a drug which targets the ERK1/2 cascade for cancer and inhibits Raf→ERK1/2 signalling in the heart (Figures 1 and 2), may be useful to reduce cardiac fibrosis in hypertensive heart disease and, although there is concomitant suppression of cardiomyocyte hypertrophy (potentially because of the reduced workload imposed by increasing cardiac fibrosis), this does not appear to be detrimental to the heart either in an acute (Figures 2 and 3) or chronic (Figures 4 and 5) setting. The data not only establish Raf kinases as viable therapeutic targets for hypertensive heart disease, but also identify an existing drug in clinical use in humans as a potential therapy.

The importance of BRAF in cancer is well-established, with a number of inhibitors in development or approved for clinical use for an increasing variety of different cancers [6,31]. Dabrafenib is a Raf inhibitor that can activate ERK1/2 via ‘Raf paradox’ signalling in cancer cells [32]. In contrast, we detected limited paradox-inducing effects of dabrafenib in primary cardiac cells or perfused hearts (Figure 1). Moreover, dabrafenib was as potent as trametinib (a MKK1/2 inhibitor also in clinical use for melanoma [33]) in inhibiting ERK1/2 activity. It is important to consider that Raf inhibitors have generally been studied in cancer cell lines, prone to proliferation and gene mutations which result in relatively fluid signalling pathways. This differs from ‘normal’ cells that are generally quiescent, possibly highly differentiated and, in the case of cardiomyocytes, terminally differentiated. In these cells, signalling pathways are potentially ‘hard-wired’ and responses cannot necessarily be extrapolated from those of cancer cells. Dabrafenib did not have significant paradox-inducing effects in the cardiac system, presumably because of a difference in the signalling pathways.

There are increasing problems with cardiotoxicity of anti-cancer drugs [25,34], including those which target the ERK1/2 cascade at the level of MKK1/2 (e.g. trametinib, cobimetinib and selumetinib). Trametinib, alone or in combination with dabrafenib, causes hypertension in up to 26% of patients with decreased ejection fraction in 7–11% of patients [25], and meta-analysis of phase II/III trials of MKK1/2 inhibitors indicate these are on-target effects [33]. In contrast, there are limited reports of cardiotoxicity with dabrafenib monotherapy. Assessment of the FDA's Adverse Event Reporting System for patients receiving either BRAF inhibitors alone or with an MKK1/2 inhibitor (2011–2019; 7712 adverse events), identified just 187 cases of cardiovascular-associated problems resulting from BRAF inhibitor monotherapy [35]. For the monotherapy, 90% of adverse events related to vemurafenib which (unlike dabrafenib) increases QTc interval [36,37].

The difference in toxicity between targeting the ERK1/2 cascade at the level of Raf kinases versus MKK1/2 may relate to alternative inputs at the level of MKK1/2. For example, Cot/Tpl2 activates MKK1/2 in some circumstances and is particularly implicated in the inflammatory response [5], and it is clear that α_1_-adrenergic agonists and oxidative stress use an alternative input to MKK1/2 in cardiomyocytes rather than Raf kinases [38,39]. These additional inputs may mean that cardiomyocytes are not compromised by Raf inhibition to the same degree as inhibiting at the level of MKK1/2. Consistent with this, dabrafenib had no significant effect on cardiac function or dimensions *in vivo* in a normotensive setting or any effect on cellular architecture or gene expression (Supplementary Tables S3 and S4; Supplementary Figure S1). In this context, given that a combination Raf plus MKK1/2 inhibitors (e.g. dabrafenib with trametinib) is the preferred cancer therapy because of the Raf paradox, patients who experience cardiotoxicity from this regime may still benefit from RAF-targeted monotherapy. Extrapolating further, could dabrafenib be cardioprotective in patients with cancer, undergoing treatments with established cardiotoxicity (e.g. anthracyclines [40])? Moreover, would a cancer environment compromise the cardioprotective effects of dabrafenib on the heart? The answer to the former may depend on the type of toxicity and the underlying mechanism by which dabrafenib exerts its effects; our data suggest the beneficial effects of dabrafenib are likely to be on longer term cardiac remodelling with fibrosis causing cardiac dysfunction rather than acute damage to the myocardium. Addressing the latter question, it seems unlikely that early stage cancer associated with dabrafenib treatment in humans would be likely to affect the cardio-protective effects of dabrafenib given that the heart itself is not prone to cancer (apart from in a very few cases) and metastatic cancers do not (as far as we are aware) invade the heart. However, the systemic effects of later stage cancer and the other treatments that patients receive could influence the responses. Clearly, these issues need to be addressed in preclinical studies and/or the clinic.

Although dabrafenib had no obvious effect in an unstressed heart, it inhibited cardiac adaptation to AngII-induced hypertension suggesting BRAF signalling is important in disease development. We examined the effects of dabrafenib on cardiac adaptation to both acute (up to 7 d) and chronic (28 d) hypertension induced by AngII. Over 3 d, the heart had compensated for the initial insult, with increased LV wall thickness and decreased LV internal diameter, with a concomitant increase in fractional shortening (Figure 2B–D). This was moderated by dabrafenib. By 7 d, the response to AngII had already changed. Fractional shortening and internal diameters had normalized, but LV wall thickness was still enhanced and this was inhibited by dabrafenib. Dabrafenib also inhibited the increase in cardiomyocyte size and both interstitial and perivascular fibrosis (Figure 3). With chronic AngII treatment, the hearts were beginning to fail, with a significant reduction in ejection fraction and increased LV wall thickness (Figure 4). This was associated with a reduction in global longitudinal and circumferential strain. Notably, dabrafenib inhibited the reduction in ejection fraction and global strain at 28 d. This was accompanied by a reduction in cardiomyocyte size and degree of fibrosis (Figure 5). Thus, our data indicate that dabrafenib may be therapeutically useful for reducing cardiac fibrosis and maintaining cardiac function in hypertensive heart disease. Clearly, further studies are required to establish if this is sustained as the heart decompensates further in response to AngII. Additional studies in other models of heart failure would also help to determine whether the effect of dabrafenib is specific to AngII-associated hypertension or if it is more generally useful in reducing cardiac fibrosis.

An important consideration in terms of potential mechanism is whether or not the effects of dabrafenib on AngII-induced maladaptive remodelling may simply be a consequence of reducing the increase in blood pressure resulting from AngII-treatment. We did not assess blood pressure in this study and this is clearly a limitation of our work. There appears to be no information to suggest that dabrafenib monotherapy reduces blood pressure in patients. Furthermore, trametinib and other MKK1/2 inhibitors promote hypertension in up to 26% of patients, irrespective of whether patients are treated with the MKK1/2 inhibitors alone or in combination with a BRAF inhibitor [25], suggesting that dabrafenib does not mitigate the hypertensive effects of trametinib. In addition, whilst histological assessment of the aortae indicated that dabrafenib reduced fibrosis resulting from AngII-treatment, the medial layer showed a similar degree of thickening with or without dabrafenib (Figure 6A). This suggests that a similar degree of hypertension was induced and the overall response of the aorta to hypertensive pressures was maintained. Nevertheless, although the weight of evidence is generally against the likelihood that dabrafenib reduced the increase in blood pressure induced by AngII-treatment, further studies are clearly required to confirm this experimentally.

Our data suggest that dabrafenib may be useful in treating cardiac fibrosis but, as with all drugs, there are considerations of on-target versus off-target effects. In our studies, dabrafenib most probably inhibits all three Raf kinases, so we cannot be certain which may be driving the ERK1/2 signal in response to AngII (Figure 2A). Assuming the signal is mediated via ERK1/2, the data are consistent with other studies indicating that ERK1/2 signalling in cardiac fibroblasts contributes to cardiac fibrosis. For example, IL11 promotes extracellular matrix production from cardiac fibroblasts via ERK1/2, acting in a post-transcriptional manner [29]. Here, it is necessary to consider the complexities associated with fibrosis. ERK1/2 could influence the rate of synthesis and processing of extracellular matrix proteins, the degree of post-translational modification and cross-linking of collagens, and also the rate of degradation. Our data indicate that at least one of the matrix metalloproteinases (TIMP1) is transcriptionally up-regulated in response to AngII and this is reduced by dabrafenib (Figure 3F). However, for these enzymes, it is more important to assess their overall activities and something which should be considered in the future. It must also be considered that dabrafenib could exert its effects via another pathway (e.g. Raf1 inhibits pro-apoptotic kinases [41,42]) or off-target effects such as inhibition of the pro-apoptotic kinase, RIPK3 [43]. Further studies with different Raf inhibitors are clearly warranted to control for these. Here, it is notable that the cancer field has moved towards development of a different class of inhibitor, Type 2 inhibitors (e.g. PLX8394) that bind exclusively to an inactive conformation of the kinase and are viewed as ‘Raf paradox’ breakers [44–46]. The cardiac effects of these drugs remain to be investigated.

Whether or not dabrafenib exerts its effects through Raf kinases themselves, it is important to consider the cellular context of the overall response. Our mRNA data suggest that there was increased inflammation in our AngII model of hypertension in mice and this was reduced by dabrafenib (Figure 3G). This remains to be confirmed at the protein level, but increased inflammation is likely to promote cardiac fibrosis [47]. Increasing cardiac fibrosis adds to the workload on the heart, potentially causing further cardiomyocyte hypertrophy. This, in itself, is likely to result in dysfunctional cardiomyocytes and cardiomyocyte death which will then lead to enhanced inflammation. As indicated in Figure 7, dabrafenib may inhibit any of these processes, possibly acting on different target enzymes. Apart from this, our data suggest dabrafenib may preserve the elasticity of the aorta (Figure 6), suggesting it could have a beneficial effect on the vasculature, which would be predicted to benefit the heart. Irrespective of mechanism, our data provide proof-of-principle that drugs such as dabrafenib may be therapeutically useful for reducing cardiac fibrosis. In this respect, there may be competition with other kinase inhibitors, including ASK1 inhibitors that have been in clinical trials for fibrotic diseases such as non-alcoholic steatohepatitis [48]. Our recent studies indicate that the ASK1 inhibitor, selonsertib, does indeed reduce cardiac fibrosis resulting from AngII-induced hypertension [22], but dabrafenib appears at least as effective.

There are, of course, limitations to this study. First, as mentioned above, further studies of the effects of dabrafenib on the increase in blood pressure induced by AngII in our mouse models are necessary. Secondly, we used rat cardiomyocytes and perfused hearts, but conducted the *in vivo* studies in mice. The reasons are technical: the rat systems are well-characterized for *ex vivo* studies, whilst mice are the preferred model for *in vivo* studies. The results were consistent across the species with dabrafenib inhibiting ERK1/2 activities in both, and suppressing cardiac hypertrophy and fibrosis *in vivo*. Thirdly, we only conducted experiments with male mice and these were juveniles. The reasons were purely practical in that it is important to first obtain proof-of-principle data for a relatively focused study. Future studies should consider assessing the effects of dabrafenib on hypertensive heart disease in female animals, in addition to older mice. The latter is particularly important given that the responses are likely to change with age and hypertension is associated with aging. A fourth consideration is the duration of our study. We only assessed the effects of a single dose of dabrafenib on AngII-induced cardiac hypertrophy over 4 weeks, during which time the hearts did not fail whereas most cancer patients are treated for much greater lengths of time with different dosages. However, we did select a dose which is relevant for humans, and cardiac dysfunction has been observed in patients receiving trametinib/dabrafenib combination therapy within 13 days [49], so our data are relevant. Nevertheless, future studies exploring different dosage regimes and more prolonged treatments would be useful. Apart from assessing potential cardiotoxicity over prolonged periods, it will be important to assess whether dabrafenib could, indeed prevent hearts from failing, and to determine if administration of the drug can prevent or even reverse cardiac fibrosis in a heart which is already diseased, a situation more relevant to the human scenario.

The final considerations for our work are the potential implications of using drugs such as dabrafenib for cardiac diseases. Dabrafenib itself does not appear to be cardiotoxic and is generally well-tolerated. There are side effects in cancer patients which are generally managed by dose reduction. The most severe effect is probably an increase in cutaneous squamous cell carcinoma (∼12% of patients) [50]. Thus, if dabrafenib were to be used as a therapy for hypertensive heart disease, it may be important to consider dosage monitoring and whether patients have a predisposition for other diseases such as cancer (in the context, perhaps, of ‘onco-cardiology’). Nevertheless, reducing cardiac fibrosis with a drug such as dabrafenib may be such a powerful tool in treating cardiac diseases that the benefits could outweigh these costs.

## Clinical perspectives

- Background: Inhibitors of the ERK1/2 cascade are used to treat cancer and some have cardiotoxic effects. Oncogenic BRAF (that activates ERK1/2) is a prime target for cancer and dabrafenib was developed as a BRAF inhibitor for melanoma. It is generally used in combination with other drugs, the combinations being cardiotoxic in some patients.
- Results summary: Dabrafenib had no overt effects on cardiac function/dimensions (assessed by echocardiography) or cardiac architecture when administered to mice *in vivo*, but inhibited cardiac fibrosis and cardiac hypertrophy in hypertensive mice.
- Clinical significance: Since dabrafenib alone did not cause cardiac dysfunction, it may be preferable for use as monotherapy (rather than in combination with other drugs that are cardiotoxic) in some patients with BRAF directed cancers. In addition, dabrafenib (and other similar inhibitors) may be therapeutically beneficial in preventing cardiac hypertrophy and fibrosis in hypertension, thus reducing heart failure development.

## Supplementary Material

Supplementary Figure S1 and Tables S1-S6

## Data Availability

All primary data are available from the corresponding author upon reasonable request. Additional data sharing information is not applicable to this study.

## Open Access

Open access for this article was enabled by the participation of University of Reading in an all-inclusive *Read & Publish* pilot with Portland Press and the Biochemical Society under a transformative agreement with JISC.

## Abbreviations

EDV: end diastolic volume
EDLVM: end diastolic left ventricular mass
EF: ejection fraction
ERK: extracellular signal-regulated kinase
ESV: end systolic volume
FS: fractional shortening
LV: left ventricular
MKK: mitogen-activated protein kinase kinase
