# Supplementary material for: The anti-cancer drug dabrafenib is not cardiotoxic and inhibits cardiac remodelling and fibrosis in a murine model of hypertension
Source: Clin Sci (Lond). 2021 Jul 23;135(14):1631–47. doi: 10.1042/CS20210192 (PMC8302807; doi:10.1042/CS20210192)
Supplement: Supplementary Figure S1 and Tables S1-S6 [file CS-2021-0192_supp.pdf]

Meijles et al. The anti-cancer drug dabrafenib is not cardiotoxic and inhibits cardiac remodelling and fibrosis in a murine model of hypertension.

Supplementary tables and figures

Supplementary Figure S1. Acute infusion of mice with dabrafenib had no significant effect on cardiac function/dimensions. C57BL/6J male mice were treated with vehicle or 3 mg/kg/d dabrafenib for 28 d. **(A)** Echocardiograms were taken at baseline, 3 d or 7 d and analysed, comparing effects of vehicle *vs* dabrafenib alone. Representative echocardiograms are shown. **(B)** Hearts were fixed and sections stained with haemotoxylin and eosin (H&E) or Masson's Trichrome as indicated. Representative sections are shown. **(C)** RNA was extracted from the hearts and mRNA expression measured by qPCR. Results are expressed relative to the means of the vehicle-treated controls. Individual data points are shown with means ± SEM.

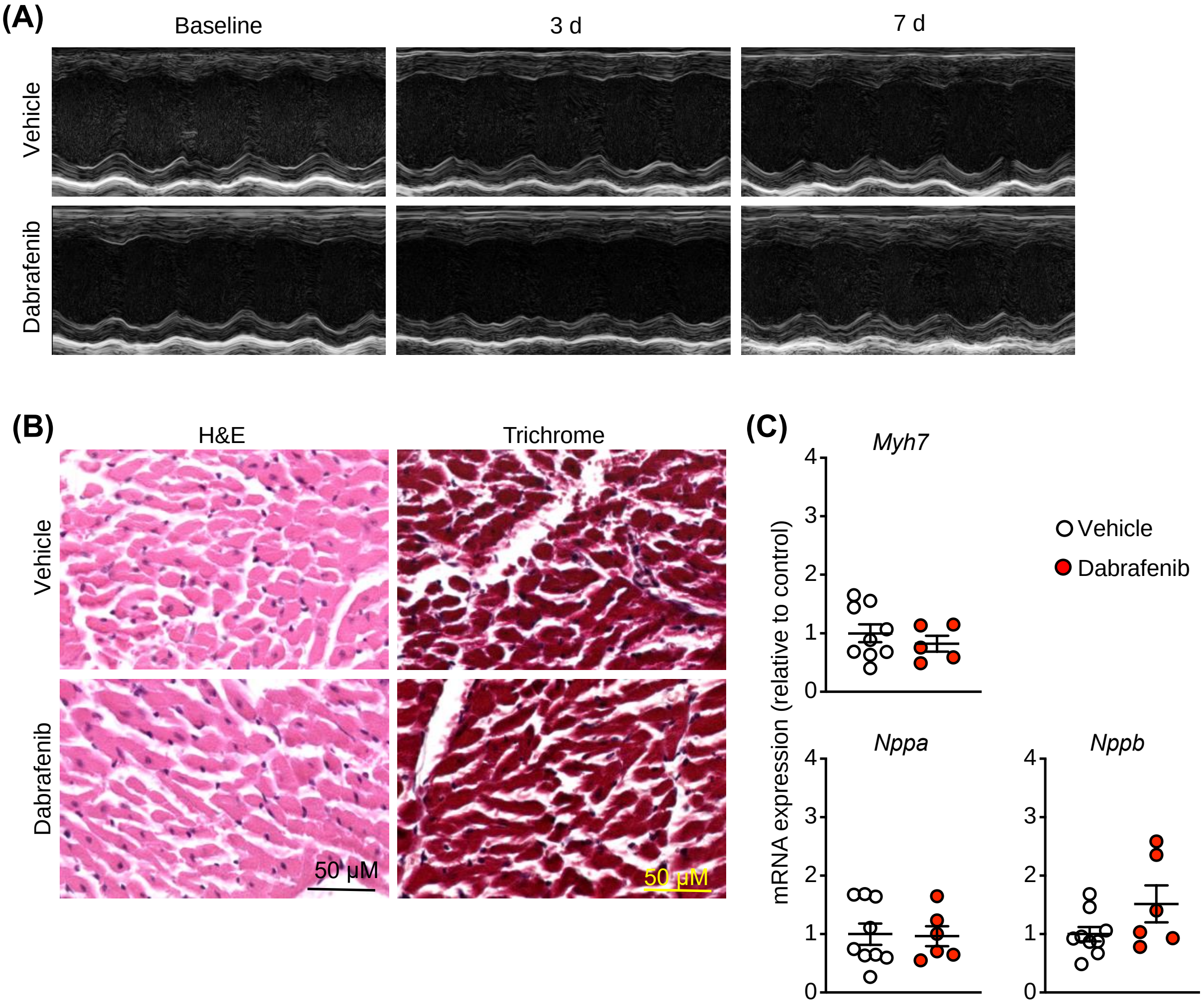

Supplementary Table S1. Mouse body weights. Body weights (BW) are in g. Weights were taken post-minipump insertion (Start) and when mice were culled (End); weights included the minipumps. p values relative to starting weight (2-way ANOVA with Holm-Sidak's post-test).

		BW:Start		BW:End		
**Study**	**Condition**	**Mean**	**SEM**	**Mean**	**SEM**	**n**
Acute AngII	Vehicle only	26.44	0.60	27.34	0.60	12
	Dabrafenib	26.26	0.40	26.87	0.45	10
	AngII	25.99	0.47	25.82	0.44	13
	Dabrafenib/AngII	25.56	0.26	25.09	0.37	10
Chronic AngII	Vehicle only	28.48	1.08	29.97*	0.84	6
	Dabrafenib	28.33	0.73	29.43*	0.72	6
	AngII	27.43	0.28	28.95*	0.39	4
	Dabrafenib/AngII	26.68	0.69	30.18*	0.57	4

*p<0.05 relative to starting weight (2-way ANOVA with Holm-Sidak post-test)

Supplementary Table S2. qPCR primer sequences

Gene Symbol	Sense Primer (5'→3')	Antisense Primer (5'→3')
Col1a1	TCGTGGCTTCTCTGGTCTC	CCGTTGAGTCCGTCTTTGC
Col4a1	CTGGCACAAAAGGGACGAG	ACGTGGCCGAGAATTTCACC
Ctgf	GCACACCGCACAGAACCA	ATGGCAGGCACAGGTCTTG
Ddr2	GCACTTGGTGAATTAATTAGAATCCTG	GGACAACTAAATGGTCCCTCCC
Fn1	AAGAGGACGTTGCAGAGCTA	AGACACTGGAGACACTGACTAA
IL1b	CAACCAACAAGTGATATTCTCCAT	GGGTGTGCCGTCTTTCATTA
IL11	TGACGGAGATCACAGTCTGGA	CGGAGGTAGGACATCAAGTCTAC
IL6	TCCATCCAGTTGCCTTCTTG	GGTCTGTTGGGAGTGGTATC
Lox	GACATTCGCTACACAGGACAT	AACACCAGGTACGGCTTTATC
Myh7	GAGATCGAGGACCTGATGG	TCATACTTCTGCTTCCACTCA
Nppa	GATGGATTTCAAGAACCTGCTAGA	CTTCCTCAGTCTGCTCACTCA
Nppb	TCCAGCAGAGACCTCAAAATTC	CAGTGCGTTACAGCCCAAA
Postn	TTCCTCTCCTGCCCTTATATGC	CCTGATCCCGACCCCTGAT
Timp1	TACGCCTACACCCCAGTCAT	GCCCGTGATGAGAAACTCTTC
Tnfa	AGCCAGGAGGGAGAACAGA	CAGTGAGTGAAAGGGACAGAAC

Supplementary Table S3. Echocardiography data: acute effects (baseline, 3 d and 7 d) of dabrafenib *in vivo*. C57BL/6J male mice were treated with vehicle, 3 mg/kg/d dabrafenib, 0.8 mg/kg/d angiotensin II (AngII) or dabrafenib/AngII for 7 d. Echocardiograms were taken at baseline (BL), 3 d or 7 d. M-mode images from short axis views were taken at the level of the papillary muscles. Data were analysed using VevoLab software. LV, left ventricular; AW, anterior wall; ID, internal diameter; PW, posterior wall; WT, wall thickness (anterior + posterior walls); d, diastole; s, systole.

	**Vehicle (n=12)**		**Dabrafenib (n=10)**		**AngII (n=13)**		**Dabrafenib/AngII (n=10)**	
**Baseline**	**Mean**	**SEM**	**Mean**	**SEM**	**Mean**	**SEM**	**Mean**	**SEM**
Heart Rate (bpm)	462	8	478	45	458	7	458	8
Ejection Fraction (%)	46.3	1.4	44.6	4.5	44.7	1.0	46.4	2.7
Fractional Shortening (%)	23.0	0.9	22.0	2.3	22.0	0.6	23.1	1.7
LVAW;d (mm)	0.762	0.013	0.779	0.075	0.776	0.015	0.787	0.015
LVAW;s (mm)	1.097	0.018	1.118	0.106	1.108	0.020	1.125	0.027
LVID;d (mm)	4.187	0.043	4.037	0.387	4.117	0.067	3.983	0.079
LVID;s (mm)	3.196	0.050	3.125	0.300	3.197	0.060	3.039	0.085
LVPW;d (mm)	0.713	0.014	0.706	0.068	0.718	0.017	0.704	0.014
LVPW;s (mm)	1.010	0.016	0.993	0.095	0.983	0.020	0.988	0.028
WT:ID; d	0.353	0.004	0.369	0.035	0.364	0.007	0.376	0.011
WT:ID; s	0.663	0.016	0.680	0.066	0.658	0.017	0.704	0.032
**3 d**	**Mean**	**SEM**	**Mean**	**SEM**	**Mean**	**SEM**	**Mean**	**SEM**
Heart Rate (bpm)	491	9	481	46	503	13	511	18
Ejection Fraction (%)	48.5	1.7	46.9	5.1	58.5	1.9	52.1	2.4
Fractional Shortening (%)	24.4	1.1	23.4	2.7	30.5	1.3	26.4	1.6
LVAW;d (mm)	0.787	0.018	0.793	0.077	0.967	0.013	0.956	0.032
LVAW;s (mm)	1.118	0.018	1.141	0.109	1.316	0.018	1.287	0.046
LVID;d (mm)	4.126	0.059	3.987	0.382	3.388	0.121	3.570	0.085
LVID;s (mm)	3.128	0.077	3.028	0.293	2.359	0.119	2.625	0.090
LVPW;d (mm)	0.706	0.011	0.739	0.078	1.053	0.050	0.872	0.049
LVPW;s (mm)	0.993	0.020	1.045	0.107	1.454	0.045	1.161	0.069
WT:ID; d	0.363	0.009	0.386	0.039	0.610	0.035	0.517	0.029
WT:ID; s	0.681	0.025	0.731	0.076	1.222	0.084	0.951	0.066
**7 d**	**Mean**	**SEM**	**Mean**	**SEM**	**Mean**	**SEM**	**Mean**	**SEM**
Heart Rate (bpm)	481	12	486	47	488	12	495	12
Ejection Fraction (%)	52.7	2.2	52.2	5.0	56.2	2.6	64.4	2.5
Fractional Shortening (%)	27.0	1.4	26.6	2.6	29.2	1.9	34.9	1.9
LVAW;d (mm)	0.806	0.019	0.819	0.081	0.936	0.022	0.948	0.043
LVAW;s (mm)	1.183	0.023	1.201	0.116	1.302	0.022	1.358	0.045
LVID;d (mm)	4.138	0.057	3.955	0.383	3.599	0.122	3.565	0.101
LVID;s (mm)	3.031	0.065	2.908	0.284	2.574	0.134	2.325	0.100
LVPW;d (mm)	0.736	0.019	0.769	0.075	0.992	0.034	0.841	0.041
LVPW;s (mm)	1.060	0.025	1.097	0.105	1.370	0.042	1.286	0.052
WT:ID; d	0.374	0.008	0.404	0.040	0.548	0.033	0.510	0.035
WT:ID; s	0.746	0.029	0.799	0.081	1.103	0.110	1.165	0.078

Supplementary Table S4. Echocardiography data: chronic effects (baseline and 28 d) of dabrafenib *in vivo*. C57BL/6J male mice were treated with vehicle or 3 mg/kg/d dabrafenib for 28 d. Echocardiograms were taken at baseline (BL) or 28 d. M-mode images from short axis views were taken at the level of the papillary muscles. Data were analysed using VevoLab software. LV, left ventricular; AW, anterior wall; ID, internal diameter; PW, posterior wall; WT, wall thickness (anterior + posterior walls); d, diastole; s, systole.

	**Baseline vehicle (n=6)**		**Baseline dabrafenib (n=6)**		**28 d vehicle (n=6)**		**28 d dabrafenib (n=6)**	
	**Mean**	**SEM**	**Mean**	**SEM**	**Mean**	**SEM**	**Mean**	**SEM**
Heart Rate (bpm)	491	13	519	11	568	22	585	8
Ejection Fraction (%)	54.7	1.9	58.2	2.1	61.6	2.6	63.7	2.4
Fractional Shortening (%)	28.3	1.3	30.5	1.3	32.8	1.8	34.3	1.8
LVAW;d (mm)	0.753	0.016	0.813	0.018	0.796	0.024	0.757	0.018
LVAW;s (mm)	1.001	0.027	1.120	0.022	1.055	0.031	1.053	0.037
LVID;d (mm)	4.208	0.117	4.129	0.178	4.026	0.150	3.994	0.112
LVID;s (mm)	3.028	0.131	2.881	0.180	2.711	0.149	2.629	0.131
LVPW;d (mm)	0.768	0.013	0.777	0.016	0.688	0.019	0.711	0.028
LVPW;s (mm)	1.065	0.020	1.090	0.011	1.079	0.034	1.104	0.031
WT:ID; d	0.363	0.010	0.388	0.018	0.370	0.011	0.369	0.014
WT:ID; s	0.699	0.041	0.781	0.044	0.798	0.045	0.831	0.049

Supplementary Table S5. Echocardiography data: chronic effects (baseline and 28 d) of dabrafenib *in vivo*. C57BL/6J male mice were treated with vehicle, 3 mg/kg/d dabrafenib, 0.8 mg/kg/d angiotensin II (AngII) or dabrafenib/AngII for 28 d. Echocardiograms were taken at baseline (BL) or 28 d. M-mode images from short axis views were taken at the level of the papillary muscles. Data were analysed using VevoLab software. LV, left ventricular; AW, anterior wall; ID, internal diameter; PW, posterior wall; WT, wall thickness (anterior + posterior walls); d, diastole; s, systole.

	**Vehicle (n=4)**		**AngII (n=4)**		**Dabrafenib/AngII (n=4)**	
**Baseline**	**Mean**	**SEM**	**Mean**	**SEM**	**Mean**	**SEM**
Heart Rate (bpm)	491	13	519	11	532	12
Ejection Fraction (%)	54.7	1.9	58.2	2.1	58.9	2.9
Fractional Shortening (%)	28.3	1.3	30.5	1.3	31.0	1.9
LVAW;d (mm)	0.753	0.016	0.813	0.018	0.764	0.027
LVAW;s (mm)	1.001	0.027	1.120	0.022	1.089	0.029
LVID;d (mm)	4.208	0.117	4.129	0.178	3.972	0.153
LVID;s (mm)	3.028	0.131	2.881	0.180	2.752	0.166
LVPW;d (mm)	0.768	0.013	0.777	0.016	0.758	0.023
LVPW;s (mm)	1.065	0.020	1.090	0.011	1.075	0.037
WT:ID; d	0.363	0.010	0.388	0.018	0.385	0.013
WT:ID; s	0.699	0.041	0.781	0.044	0.807	0.059
**28 d**	**Mean**	**SEM**	**Mean**	**SEM**	**Mean**	**SEM**
Heart Rate (bpm)	511	19	557	24	551	16
Ejection Fraction (%)	59.6	2.1	47.1	3.8	62.1	2.1
Fractional Shortening (%)	31.6	1.4	23.6	2.3	33.1	1.4
LVAW;d (mm)	0.787	0.006	0.942	0.029	0.784	0.012
LVAW;s (mm)	1.072	0.015	1.192	0.043	1.093	0.009
LVID;d (mm)	4.284	0.086	4.210	0.202	3.899	0.126
LVID;s (mm)	2.929	0.056	3.214	0.173	2.613	0.130
LVPW;d (mm)	0.807	0.018	0.907	0.015	0.739	0.017
LVPW;s (mm)	1.182	0.021	1.143	0.042	1.081	0.026
WT:ID; d	0.373	0.010	0.443	0.019	0.397	0.010
WT:ID; s	0.771	0.013	0.735	0.043	0.846	0.042

Supplementary Table S6. Echocardiography data: measurements of aortic diameter for effects of dabrafenib *in vivo*. C57BL/6J male mice were treated with vehicle, 3 mg/kg/d dabrafenib, 0.8 mg/kg/d angiotensin II (AngII) or dabrafenib/AngII for 7 or 28 d. Echocardiograms were taken at baseline (BL), 7 d or 28 d. B-mode images were taken of the ascending aorta and the internal aortic diameter measured at the end of cardiac systole (s) or during diastole (d). Data were analysed using VevoLab software using callipers.

	**Vehicle**		**Dabrafenib**		**AngII**		**Dabrafenib/AngII**	
**7 d (n=8)**	**Mean**	**SEM**	**Mean**	**SEM**	**Mean**	**SEM**	**Mean**	**SEM**
Aortic diameter; s (mm)	1.450	0.026	1.446	0.023	1.490	0.046	1.480	0.040
Aortic diameter; d (mm)	1.227	0.036	1.221	0.019	1.336	0.062	1.292	0.037
Aortic diameter; s:d ratio	1.184	0.015	1.186	0.029	1.122	0.027	1.146	0.008
**28 d (n=4)**	**Mean**	**SEM**	**Mean**	**SEM**	**Mean**	**SEM**	**Mean**	**SEM**
Aortic diameter; s (mm)	1.585	0.004	1.523	0.031	1.699	0.036	1.559	0.044
Aortic diameter; d (mm)	1.352	0.011	1.262	0.038	1.605	0.058	1.368	0.073
Aortic diameter; s:d ratio	1.173	0.011	1.209	0.018	1.062	0.021	1.147	0.033
